# Hesperidin, a Bioflavonoid in Cancer Therapy: A Review for a Mechanism of Action through the Modulation of Cell Signaling Pathways

**DOI:** 10.3390/molecules28135152

**Published:** 2023-06-30

**Authors:** Arshad Husain Rahmani, Ali Yousif Babiker, Shehwaz Anwar

**Affiliations:** Department of Medical Laboratories, College of Applied Medical Sciences, Qassim University, Buraydah 51542, Saudi Arabia

**Keywords:** hesperidin, apoptosis, inflammation, signal transduction, cancer therapy

## Abstract

Cancer represents one of the most frequent causes of death in the world. The current therapeutic options, including radiation therapy and chemotherapy, have various adverse effects on patients’ health. In this vista, the bioactive ingredient of natural products plays a vital role in disease management via the inhibition and activation of biological processes such as oxidative stress, inflammation, and cell signaling molecules. Although natural products are not a substitute for medicine, they can be effective adjuvants or a type of supporting therapy. Hesperidin, a flavonoid commonly found in citrus fruits, with its potential antioxidant, anti-inflammatory, and hepatoprotective properties, and cardio-preventive factor for disease prevention, is well-known. Furthermore, its anticancer potential has been suggested to be a promising alternative in cancer treatment or management through the modulation of signal transduction pathways, which includes apoptosis, cell cycle, angiogenesis, ERK/MAPK, signal transducer, and the activator of transcription and other cell signaling molecules. Moreover, its role in the synergistic effects with anticancer drugs and other natural compounds has been described properly. The present article describes how hesperidin affects various cancers by modulating the various cell signaling pathways.

## 1. Introduction

Cancer represents one of the major causes of mortality around the world. According to cancer statistics, there will soon be a total of 1,918,030 new cases of cancer and 609,360 cancer-related fatalities [1]. The therapeutic choices against cancer include radiation therapy, chemotherapy surgical procedure, and target and gene therapy [2]. The growth and progression of cancer are largely attributed to exogenous variables such as nutrition, the environment, tobacco, chemicals, lifestyle, obesity, diet, and infectious organisms, as well as intrinsic factors such as inherited mutations, genetic predisposition, and immune conditions [3,4,5]. The current modes of treatment cause adverse effects, in addition to being expensive. 

Natural products and their bioactive molecules play a significant role in disease management, with lesser adverse effects. Studies conducted in the past have shown that natural substances or their key components can improve health by decreasing oxidative stress and inflammation [6,7,8], managing diabetes [9], and contributing to the regulation of cell signaling pathways [10,11,12,13].

**Figure 1:**
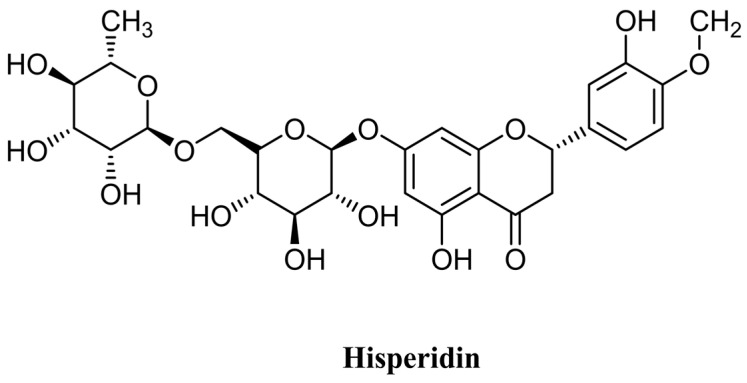
The chemical structure of hesperidin.

Hesperidin is a flavonoid that comes under the flavanone group [14] (Figure 1), which is among the most typical and widely distributed groups of plant phenol compounds. Citrus fruits are a rich source of hesperidin [15,16]. In addition to the citrus species, hesperidin is present in plant genera such as Fabaceae [17], Betulaceae [18], and Lamiaceae [19]. Its role in health management has been noted due to its antioxidant, anti-inflammatory, and anti-cancerous properties, as well as its ability to inhibit other types of pathogenesis. 

Supplemental hesperidin has been reported to improve the senescence of cells, the survival of cells, the activity of mitochondria, and the kinetics of proliferation. Hesperidin was further reported to improve the antioxidant potential of superoxide dismutases in human chondrocytes [20]. In addition, the anticancer potential of hesperidin has been described through different mechanisms of action [21]. This review describes how hesperidin regulates numerous cell signaling pathways that influence different kinds of cancer. Moreover, its synergistic effects with other natural compounds or anticancer compounds are described accordingly.

## 2. Potential Mechanisms of Hesperidin in the Preventative and Therapeutic Management of Cancer

**Figure 2:**
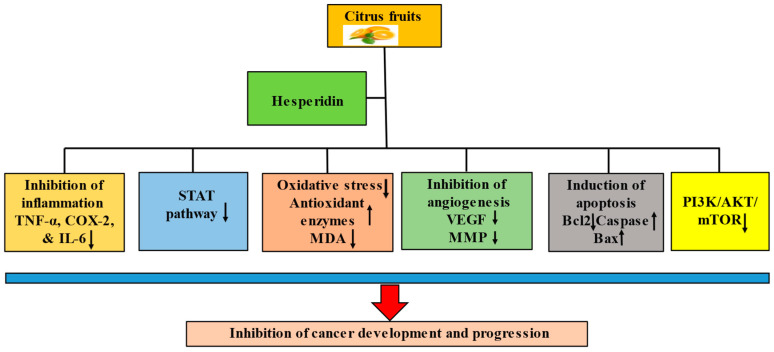
The mechanism of hesperidin in cancer prevention.

Hesperidin has been demonstrated to have anticancerous potential due to its ability to regulate various cell signaling molecules. The anticancer potential of hesperidin has been documented through the modulation of inflammation, cell cycle arrest, apoptosis, angiogenesis, and cell signaling pathways (Figure 2).

### 2.1. Inflammation

Approximately 10% of cancer cases are attributed to germline mutations; the majority of cancers are caused by acquired factors, including environmental factors, which are closely linked with chronic inflammation [22]. The decrease in inflammatory response might result in an increased risk of cancer progression and development because inflammation serves as the first line of defense against various carcinogens [23,24]. Additionally, chronic inflammation frequently results in the elevation of pro-inflammatory mediators like cyclooxygenase-2 and nitric oxide synthase, which are responsible for the elevated levels of prostaglandins and nitric oxide [25,26]. 

Hesperidin has been shown to have potent anti-inflammatory potential, as confirmed by different studies. In the mouse model of asthma with allergies, hesperidin was found to inhibit the production of inflammatory mediators. The administration of hesperidin was shown to substantially lower Th2 cytokines as well as the percentage of infiltrating inflammatory cells in the bronchoalveolar lavage fluid in comparison with mice that were exposed to ovalbumin. Hesperidin also reduced the serum levels of ovalbumin-specific IgE [27]. 

Hesperidin treatment suppressed the production of nitrogen dioxide, prostaglandin E2 (PGE2), and the expression of the nitric oxide synthase (iNOS) protein. Hence, hesperidin is now known to be a cyclooxygenase-2 and iNOS inhibitor, which could account for its antitumorigenic as well as anti-inflammatory properties [28]. 

The effectiveness of hesperidin on renal carcinogenesis induced by N-nitrosodiethylamine and iron nitrilotriacetate (Fe-NTA) was studied. The results showed that hesperidin improved renal function and further reduced lipid peroxidation induced by N,N-diethylnitrosamine (DEN) and Fe-NTA. Additionally, it was discovered that hesperidin decreased PGE2 levels and downregulated the expression of cyclooxygenase-2 (COX-2) and vascular endothelial growth factor (VEGF). These findings imply that hesperidin could serve as an effective inhibitor of renal cancer due to its capacity to reduce oxidative stress and ability to interfere with the COX-2/PGE2 pathway [29] (Table 1).

### 2.2. Oxidative Stress

An imbalanced production and depletion of reactive oxygen species (ROS) and other free radicals due to a dysfunctional antioxidant defense system is known as oxidative stress [30]. In general terms, reactive oxygen species (ROS) and free radicals are produced by properly functioning cellular metabolism, and these free radicals and ROS play a significant role in cell signaling mechanisms [31]. In addition, oxidative stress has been reported to be involved in the development of various diseases, including cancer [32,33,34]. In addition, cancer cells have deregulated redox homeostasis. ROS are reported to be pro-tumorigenic, but high concentrations of ROS are found to be cytotoxic [35]. 

Due to their ability to reduce free radical production and oxidative stress, natural products or bioactive compounds are known to restrict pathogenesis [36]. A study based on lung cancer reported that the administration of benzo(α)pyrene (b[α]p) to mice caused the elevation of lipid peroxidation, aryl hydrocarbon hydroxylase, and lung-specific tumor marker carcinoembryonic antigen. In addition, it was also reported to enhance alanine transaminase. In contrast, b[α]p was found to reduce the degrees of tissue antioxidants, like catalase, glutathione peroxidase, superoxide dismutase, and glutathione. However, the supplementation of hesperidin was involved in the restoration of normal values for these parameters. It indicates its strong anticancer ability against lung cancer. Additionally, these results support the ability of hesperidin to protect mice from developing lung cancer brought on by different chemicals [37]. 

Hesperidin was further shown to suppress Michigan Cancer Foundation-7 (MCF-7) cells growth in a concentration-dependent manner. In MCF-7 cells subjected to hesperidin, glutathione (GSH) was reported to be depleted. However, lactate dehydrogenase (LDH) was documented to be elevated. The stimulation of apoptosis in MCF-7 cells may be caused by a reduction in GSH [38]. The therapeutic role of hesperidin was further investigated in rats with breast cancer induced by 7,12-dimethybenz(a)anthracene. This study looked at the activities of lipid peroxidation as well as multiple marker enzymes bound to membrane. Hesperidin was administered orally every day in the abovementioned rats, and hesperidin caused a significant reduction in the degrees of lipid peroxidation and marker enzyme bound to the membrane. Furthermore, the administration of hesperidin significantly restored the normal functioning of an attached membrane crucial marker in the serum of both controls as well as trial rats with breast cancer [39] (Table 1). 

In the kidney and liver homogenates of rats subjected to carbon tetra chloride (CCl_4_), the levels of TBARS (thiobarbituric acid reactive substances) were significantly elevated. However, the concentrations of superoxide dismutase, glutathione content, and catalase were documented to be decreased. In contrast, hesperidin (200 mg/kg) substantially reduced the adverse consequences of carbon tetrachloride. Oxidative stress caused by carbon tetrachloride in the liver and kidneys of rats was decreased by the administration of hesperidin. This study established the beneficial effects of hesperidin [40]. 

The testicular activities of glutathione-S-transferase, lactate dehydrogenase, and superoxide dismutase were all reported to be decreased by the administration of polyaromatic hydrocarbons. Additionally, it decreased glutathione levels in the testicles while increasing malondialdehyde (MDA) levels. However, the administration of hesperidin repaired all histological and biochemical changes brought on by the b[α]p challenge [41].

The MDA and nitric oxide levels were noticeably greater in the DEN-treated group than in the untreated group or control. Additionally, the activities of catalase (CAT) and glutathione peroxidase (GPx) in the DEN group were considerably reduced. Surprisingly, the results showed that the nitric oxide (NO) and MDA levels were crucially lower in the hesperidin plus DEN group in comparison to those found in the DEN group. Likewise, compared to the DEN group, the hesperidin plus DEN group displayed substantially greater CAT and GPx functions [42].

### 2.3. Apoptosis

It is understood that apoptosis is a crucial mechanism for development as well as tightly regulated cell death [43]. Natural products or bioactive molecules, including hesperidin, have been shown to be implicated in cancer management through the induction of apoptosis. The consequences of hesperidin for apoptosis among individuals with cervical cancer were investigated. In addition, DNA fragmentation and increased nuclear condensation in cervical cancer HeLa cells were suggested to be indicators of hesperidin-initiated apoptosis. Furthermore, apoptosis stimulated by hesperidin treatment in cancer cells took place via a mechanism dependent on caspase, which appeared to be downstream of the endoplasmic reticulum stress pathway, as indicated by increased levels of GADD153/CHOP and GRP78. Additionally, hesperidin supported the formation of ROS as well as a reduction in the membrane potential of mitochondria. Further, it induced the mobilization of intracellular Ca^2+^, higher discharge of cytochrome C, and an apoptosis-inducing factor via mitochondria. It was further reported to encourage the activation of capase-3. Hesperidin was found to inhibit cell cycle progression and apoptosis by connecting endoplasmic reticulum stress pathways [44] (Table 1). Additionally, hesperidin caused apoptotic signaling, leading to the breaking down of the BH3 interacting domain death agonist (Bid), caspase-3 (CASP3), and poly (ADP-ribose) polymerase (PARP) as well as the elevated levels of Bcl-2-associated X protein (Bax). In addition, it caused a reduction in Bcl-xl (B-cell lymphoma-extra large) [45]. 

The apoptosis as well as viability of lung cancer H460 and A549 cells were investigated, followed by the administration of hesperidin (1 as well as 2.5 µM). The results established that hesperidin administration prevented the proliferation of H460 as well as A549 cells and induced apoptosis in a dose-dependent way [46]. A flow cytometric apoptosis assay was carried out to identify the molecular mechanisms linked with the reduced viability of the A549 lung cancer cell line by hesperidin. According to the findings, the apoptotic rate in the control group was less than 5%. Comparing the rates of apoptosis in the groups administrated various amounts of hesperidin for various lengths of time to the ones in the control group, it was found that the outcome was both time- and dose-dependent [47]. According to research on the expression of genes that control apoptosis, hesperidin therapy decreased the expression of B-cell CLL/lymphoma 2 (BCL2) mRNA and increased the expression of BCL2-associated X protein. With hesperidin administration, the expression and functions of the primary apoptotic factor CASP3 were both significantly increased. Hesperidin decreased the protein expression of pro-CASP3 and increased the level of active CASP3. These results suggest that hesperidin may activate CASP3 to encourage apoptosis among human colon cancer cells [48] (Table 1). 

Therapy with hesperidin caused apoptosis in gastric cancer AGS cell lines, which were marked with nuclear condensation and cell shrinking. Hesperidin also increased intracellular ROS and caused mitochondrial apoptosis in these cancer cells. The pre-treatment of gastric cancer AGS cells with N-acetyl cysteine (NAC) stopped the apoptosis that was induced by hesperidin. Western blotting results showed that p-JNK (Jun N-terminal kinase), BCL2-associated agonist of cell death (Bad), CASP3, p-p38, and PARP expression was increased. However, the expression of antiapoptotic proteins Bcl-2 and phospho-extracellular signal-related kinase (p-ERK) was decreased. This indicated the fact that hesperidin triggered the MAP kinase (MAPK) signaling cascade and apoptosis dependent on mitochondria in AGS gastric cancer cells. Human gastric cancer cells are encouraged to undergo apoptosis by hesperidin [49] (Table 1).

### 2.4. Cell Cycle

Eukaryotic cells have mechanisms called cell cycle checkpoints that allow the investigation of stress-induced cellular damage and the repair of it [50]. The management of the cell cycle is considered as important step in the prevention of cancer. Hesperidin and other bioactive substances have a significant impact on the prevention of cancer. According to flow cytometry-based results, hesperidin has the ability to stop the advancement of the regular cell cycle in MG-63 cell lines of human osteosarcoma. The findings demonstrated that hesperidin directed to the G2/M phase cell cycle arrest at increasing doses. The proportions of G2/M cells were increased from 29.2% in untreated cells to 35.1, 46.3, and 72.4% in cells exposed to 5, 50, and 150 µM of hesperidin, respectively. When the concentration of hesperidin rose from 0 to 150 µM, the G0/G1 cell population decreased concurrently [51] (Table 1). 

Using flow cytometry, the consequences of hesperidin treatment on the propagation of HeLa cells during the cell cycle were investigated to understand the mechanism by which hesperidin inhibits cell growth. Hesperidin treatment caused cell cycle arrest in cancer cells at the G0/G1 stage in a way that was dose-dependent in HeLa cells from the cervix. Hesperidin treatment at concentrations of 160, 80, and 40 µM raised the number of cells in the G0/G1 stage by 1.71, 1.43, and 1.22 times, respectively, compared to control cells. A concentration-dependent cell cycle arrest caused by hesperidin was seen in these cancer cells [44] (Table 1). 

Hesperidin may have an impact on how lung cancer A549 cells are distributed throughout the cell cycle. In comparison to the control group, the groups subjected to different doses of hesperidin at various timings showed a meaningful increase in the proportion of cells present in the G0/G1 stage, suggesting the arrest of cell cycle or growth in the G0/G1 stage. Additionally, hesperidin decreased the expression levels of cyclin D1, which is a cell cycle regulating protein, while increasing the relative expression levels of p53 and p21. Comparative quantitative analysis revealed significant differences from the control. According to these findings, hesperidin led to G0/G1 phase hold or arrest in A549 cells by modulating the proportionate protein expression linked to the cell cycle [47]. 

At the same dose, hesperidin treatment resulted in increased nuclear condensation and ROS production. A dose-dependent increase in the activation of CASP3 and the arrest of the cell cycle at the G2/M stage was also observed. These findings indicate that hesperidin might be able to work as an anticancer substance for the treatment of gall bladder carcinoma [52] (Table 1).

### 2.5. STAT3 (Signal Transducer and Activator of Transcription)

STAT3 has been reported to be implicated in the modulation of various important biological processes, such as cell proliferation, angiogenesis, invasion, differentiation, apoptosis, inflammation, and metastasis [53,54]. The modified STAT3 action has been implicated in the progression of various hematological as well as solid tumors [55,56,57]. Interferon-gamma induced oral cancer cell lines (HN6 and HN15) to express the programmed cell death ligand1 protein by phosphorylating STAT1 and STAT3. Hesperidin also effectively suppressed phosphorylated STAT1 and STAT3 to reduce that initiation. To sum up, hesperidin used its anticancer properties with oral cancer cells by inhibiting the expression of programmed cell death ligand 1 (PD-L1) by inactivating the STAT1 and STAT3 signaling molecules [58]. Another study revealed that genes for STAT3, STAT2, and STAT1 were considerably downregulated following the administration of hesperidin. Hesperidin was predicted to slow the growth of endometrial carcinoma cells by downregulating STAT3 activation [59].

### 2.6. PI3K/AKT Pathway

One of the most important intracellular pathways, phosphoinositide 3-kinase (PI3K)/AKT/mammalian target of rapamycin (mTOR), signals control metabolism, angiogenesis, cell growth, and motility [60,61]. Tumor growth and treatment resistance are both influenced by the activation of the PI3K/AKT/mTOR pathway [61]. The concentrations of PI3K and phospho-Akt proteins were reported to be considerably higher in the group receiving only DEN compared to the control group, according to a key study finding. Additionally, the concentrations of the same proteins in the group receiving hesperidin as well as the DEN group were very low in comparison with the group treated with DEN only. These findings showed that hesperidin prevented the development of hepatocarcinogenesis brought on by DEN by inhibiting the PI3K/Akt signaling pathway. Hesperidin administration also decreased the upregulation of the expression of Akt, PI3K, and cyclin-dependent kinase-2 (CDK-2) proteins brought on by DEN and successfully retained the liver tissues’ integrity against the onset of HCC (hepatocellular carcinoma) [42].

### 2.7. Angiogenesis 

It was anticipated that antiangiogenesis monotherapies would not be effective in treating tumors in people since angiogenesis inhibitors prevent the development of new blood vessels, which would slow tumor growth but leave it unregulated. [62,63]. Bioactive compounds, including hesperidin, play a role in cancer prevention through the inhibition of angiogenesis. Hesperidin was found to lower PGE2 levels and downregulate the expression of vascular endothelial growth factor as well as COX-2 according to a study on renal carcinogenesis. Additionally, a small quantity of VEGF immunostaining was seen in the renal cells of control rats in an immunohistochemical analysis of VEGF. However, both DEN-initiated and Fe-NTA showed significant VEGF immunostaining among the renal cells. Low-dose hesperidin treatment (100 mg/kg b. wt) partially decreases VEGF immunostaining. Rats administered an increased concentration of hesperidin (200 mg/kg b.wt.) showed decreased VEGF expression [29]. In untreated groups, Ehrlich tumor cells showed an average localization of VEGF. All cells in DOX-treated groups, either individually or when combined with hesperidin, demonstrated a noticeable increase in VEGF expression. In the Solid Ehrlich carcinoma (SEC) group, hesperidin therapy reduced the level of expression of VEGF compared to the control group [64].

### 2.8. Activating Protein-1

Activating protein-1 is a dimeric protein, and the development of multiple tumor cell lines is strongly inhibited through activating protein-1 decoys or dominant negative c-Jun mutants, demonstrating the importance of activating protein-1 activity for tumorigenesis [65,66,67]. In a significant study, the promoter activity of genes, including activating protein-1 (AP-1) and nuclear factor kappa B (NF-kB), was examined by a luciferase assay to determine whether the transcriptional activities of these genes are regulated by 12-O-tetradecanoylphorbol-13-acetate (TPA). The promoter region of NF-kB or AP-1 in a genomic fragment was subcloned into the pGL3-basic vector and introduced into HepG2 cells. The promoter activity was then evaluated after the cells were exposed to TPA with or without hesperidin. Hesperidin inhibited the TPA-induced nearly 4-fold increase in NF-kB promoter activity in pGL3-basic-transfected cells in a concentration-dependent manner.

The NF-kB promoter was stimulated almost four times over the activity in cells transfected with pGL3-basic as a result of TPA treatment, and this was prevented by the administration of hesperidin in a dose-dependent manner. Additionally, in the reaction to TPA, the AP-1 promoter was activated about five times more than it was in cells that had the pGL3-basic transfection. Additionally, the administration of two concentrations of hesperidin (100 µM and 50 µM) prevented TPA-induced protein–DNA binding specific to both NF-kB as well as AP-1 in comparison with cells given only TPA [68] (Table 1).

### 2.9. ERK1/2 MAPK Pathway

The expression of ERK1/2 was examined to determine whether mitogen-activated protein kinases (MAPKs) may be involved in HepG2 cell death brought on by hesperidin. Hesperidin was discovered to substantially raise p ERK1/2 levels of protein while substantially reducing total ERK1/2 protein levels, demonstrating that hesperidin leads to paraptosis by stimulating ERK1/2. In order to further quantify the functional importance of MAPK, modifications to ERK protein levels were examined in either the presence or absence of U0126 (a particular ERK inhibitor). ERK1/2 phosphorylation was noticeably hindered by U0126, which also reversed the induced effects of pERK1/2. HepG2 cells exposed to 10 µM U0126 and 1 mM hesperidin were examined for their cellular ultrastructure using transmission electron microscopy (TEM) to investigate the role of ERK1/2 in hesperidin-linked paraptosis. It was found that U0126 stops HepG2 cells exposed to hesperidin alone from exhibiting widespread vacuolation of the cytoplasm as well as swelling of the endoplasmic reticulum and/or mitochondria. According to these findings, HepG2 cells that have been exposed to hesperidin undergo paraptosis [69] (Table 1).

**Table 1:** The mechanism of action of hesperidin in cancer prevention.

Action	Cancer	Mechanism	Refs.
Inflammation	Renal	The amount of PGE2 and COX-2 expression was both found to be downregulated by hesperidin.	[29]
Oxidative stress	Breast	The daily oral treatment of hesperidin in rats with breast cancer confirmed a significant decline in membrane-bound marker enzymes and levels of lipid peroxidation.	[39]
Apoptosis	Cervix	Hesperidin-initiated apoptosis in cervical cancer cells was characterized by a raised proportion of nuclear condensation as well as fragmentation of DNA. In addition, an enhanced percentage of GADD153/CHOP as well as GRP78 designated apoptosis caused by hesperidin in cancer cells.	[44]
	Lung	Treatment with hesperidin meaningfully prevented the proliferation of cancer cells in the lung and suggestively encouraged apoptosis in a manner that is dependent on dose.	[46]
		The rates of apoptosis in various groups with different doses of hesperidin for different time periods were raised.	[47]
	Colon	Hesperidin up-regulated the level of active CASP3 and down-regulated the protein expression of pro-CASP3.	[48]
	Gastric	Hesperidin caused apoptosis linked to mitochondria in gastric cancer cells and enhanced the percentage of intracellular ROS. The prior administration of gastric cancer AGS cells using NAC prevented the apoptosis brought on by hesperidin.	[49]
Cell cycle	Bone	Raising hesperidin concentrations leads to the arrest of the G2/M stage of the cell cycle.	[51]
	Cervix	Raising the concentrations of hesperidin leads to the arrest of the G0/G1 phase of the cell cycle.	[44]
	Lung	In contrast to the control group, the groups that received different doses of hesperidin at various timings demonstrated a significant rise in the percentage of cells in the G0/G1 phase, indicating cell cycle/growth arrest at the G0/G1 stage.	[47]
	Gall bladder	Caspase-3 activation as well as arrest of the cell cycle at the G2/M phase were increased in a way that was dependent on concentration.	[52]
Signal transducer and activator of transcription	Oral	Hesperidin inhibited PD-L1 expression by inactivating the STAT1 and STAT3 signaling molecules in oral cancer cells.	[58]
	Endometrium	Hesperidin reduces the growth of the endometrial carcinoma cells via downregulation of STAT3 activation	[59]
PI3K/Akt	Liver	By inhibiting the PI3K/Akt signaling pathway, hesperidin provided protection from hepatocarcinogenesis caused by DEN.	[42]
Angiogenesis	Renal	Treatment with low-dose hesperidin decreases VEGF immunostaining partly. High-dose hesperidin-treated rats further decreased the expression of VEGF.	[29]
Activating protein-1	Liver	Hesperidin therapy inhibited TPA-induced NF-kB- and AP-1-specific DNA–protein interactions in contrast with TPA-induced cells.	[68]
ERK1/2		In HepG2 cells treated with hesperidin alone, it has been found that U0126 inhibits the widespread cytoplasmic vacuolation and the swelling of the endoplasmic reticulum and/or mitochondria. These findings suggest that hesperidin-induced paraptosis in HepG2 cells involves ERK1/2.	[69]

## 3. Role of Hesperidin as Both a Cancer Preventative and Curative Agent 

**Figure 3:**
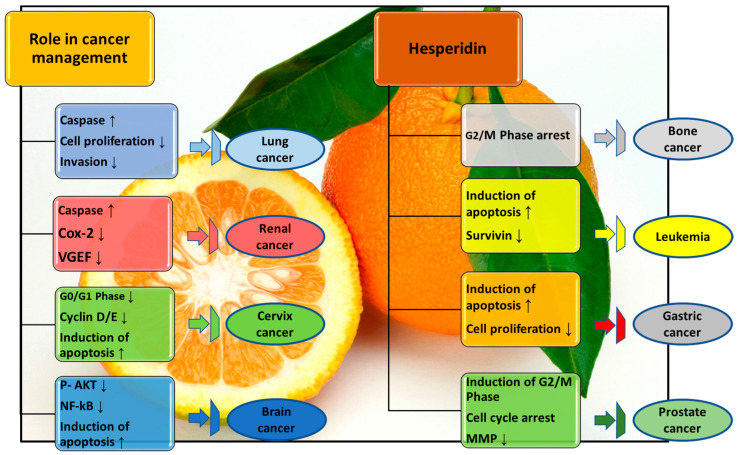
Role of hesperidin in the management of different types of cancer.

The ability of hesperidin to modify the signal transduction pathway is well-known for its application in the therapeutic management of cancer (Figure 3). The specific functions of hesperidin as both a cancer preventative and therapeutic agent are as follows:

### 3.1. Lung Cancer

Non-small-cell lung cancer (NSCLC), which makes up approximately 85 percent of instances, has become the most prevalent cancer worldwide [70]. Radiotherapy, surgical resection, and chemotherapy are the main treatment modalities for non-small-cell lung cancer, but the chances of survival for individuals who have such procedures are very low [71]. A natural compound-based study reported that natural compounds have the ability to inhibit cancer initiation and growth. Hesperidin treatment meaningfully decreased the expression of zinc finger E-box binding homeobox 3 mRNA (ZEB2 mRNA) as well as protein in a concentration-dependent manner. These results indicate that hesperidin inhibits ZEB2 expression. Furthermore, the MTT (3-[4,5-dimethylthiazol-2-yl]-2,5 diphenyl tetrazolium bromide) assay as well as flow cytometry analysis results showed that hesperidin administration meaningfully prevented the proliferation of A549 and H460 cells. Further, it meaningfully indorsed apoptosis in a dose-dependent way [46]. Lung cancer cells (A549 and NCI-H358) that were administered with hesperidin showed a concentration- and time-dependent decrease in cell proliferation. In addition, these tested cells showed increased CASP3 as well as other apoptotic activities and decreased mitochondrial membrane potential activities. These findings indicate that hesperidin inhibits NSCLC cell growth in vitro by altering immune response-related pathways that influence apoptosis [72] (Table 2). Hesperidin’s anticancer potential in case of cell proliferation and promotion of apoptosis associated with human lung cancer was investigated in addition to the mechanisms underlying these effects. Hesperidin treatment pointedly decreased the cell viability in a concentration- and time-dependent way. Hesperidin-treated cells also significantly reduced the expression of the proteins cellular myelocytomatosis (c-myc), proliferating cell nuclear antigen (PCNA), and β-catenin, which inhibited cell proliferation. Hesperidin significantly increased the levels of the tumor suppressor proteins p53 and p21, which consequently suppressed cyclin D and cyclin-dependent kinase 4 from being expressed. Further research has shown that the Bcl-2/Bax ratio is influenced directly by hesperidin therapy by reestablishing tumor suppressor proteins. Overall, these results support the anticancer potential of hesperidin in human lung cancer cells, which is caused by activating p53, which inhibits cell growth and activates mitochondrial-dependent apoptosis [73] (Table 2). Hesperidin demonstrated a dose-dependent implication in the suppression of cells of lung cancer (A549 and NCI-H460) proliferating activity as well as invasion. Additionally, hesperidin promoted the apoptosis of exhausted cancer cells. Hesperidin elevated p53 expression, discouraged p53 from interacting with MDMX (murine double minute X), and had a cancer-prevention effect [74] (Table 2).

### 3.2. Breast Cancer

The leading cause of mortality for women worldwide is breast cancer, which is also a cancer that is most frequently diagnosed in women in developed nations [75,76]. Annually, 2 million new cases and 500,000 deaths due to breast cancer are documented around the world [77]. The antitumor properties of hesperidin were investigated separately and in combination with doxorubicin in breast cancer patients. Compared to the animal groups that had been exposed to 7,12-dimethylbenz(a)anthracene (DMBA), the hesperidin-pretreated animal groups showed decreased tumor volume, decreased tumor incidence, and greater survival rates. Comparing doxorubicin-treated animals to those that had been pretreated with hesperidin, the latter group showed a significant decrease in malondialdehyde (MDA) in addition to elevated concentrations of glutathione and inflammatory markers. In comparison to DMBA-induced tumors, histopathology and Ki67 expression revealed that pretreatment with hesperidin enhanced tumor management [78] (Table 2). 

The MTT assay was used to determine whether hesperidin was cytotoxic to MDA-MB231 cells. According to the findings, hesperidin considerably reduced cell viability in comparison to the control group. In addition, PD-L1 expression along with the signaling pathway proteins p-p65, p-Akt, and p-ER were noticeably inhibited by hesperidin treatment (10 to 50 µM) in contrast with the control group. The findings indicate that PD-L1 promotes the progression of breast cancer, whereas hesperidin inhibits it by inhibiting the NF-κB and Akt signaling pathways [79] (Table 2). Animals exposed to the genotoxin DMBA showed notable modifications in the drug metabolizing enzymes and a significant decline in the status of antioxidants. Surprisingly, animals administered hesperidin had their altered levels meaningfully restored to close to normal through increased concentrations of internal antioxidants, the stimulation of phase II enzymes, and the modulation of phase I enzyme levels [80]. Both hesperidin and luteolin significantly increased the number of cells showing apoptosis in both G0/G1 as well as sub-G1 cell cycle phases. In addition, both drugs caused apoptosis by both pathways, including intrinsic as well as extrinsic. The viability of cells was decreased in a way that was dependent on both dose and time. Additionally, the downregulation of an antiapoptotic gene, i.e., Bcl-2, took place. However, pro-apoptotic Bax was upregulated. Additionally, the turning on (expression) of microRNAs, including miR-34a and 16, was significantly upregulated. In contrast, the expression of miR-21 was significantly downregulated within MCF-7 by hesperidin and luteolin [81].

### 3.3. Cervix Cancer

Treatment with hesperidin resulted in a dose-dependent up-regulation of p27 and a down-regulation of the c-Jun activation domain-binding protein-1 (Jab1) gene. The excessive formation of ROS and CASP3 activation, which further results in the induction of apoptosis, may cause these gene intonations. Hoechst staining, as well as cell cycle analysis, revealed a rise in apoptotic cells. These findings strongly suggest that the therapeutic target of hesperidin causes apoptosis and the inhibition of cell growth within HeLa cells is Jab1 [82]. Hesperidin inhibited HeLa cell proliferation in a concentration- and time-dependent manner. In this cancer cell population, hesperidin-induced apoptosis was characterized by increased DNA fragmentation and nuclear condensation. In addition, apoptosis caused by hesperidin administration in HeLa cells that was marked with elevated GRP78 in addition to raised GADD153/CHOP levels took place in a caspase-dependent pathway that appeared to be a downstream component of the stress mechanism of the endoplasmic reticulum. These two proteins are indicators of stress in the endoplasmic reticulum in the body. Additionally, hesperidin supported the production of reactive oxygen species, reduction in the potential of the mitochondrial membrane, the mobilization of intracellular Ca^2+^, the greater discharge of cytochrome c and a factor that induces apoptosis from the mitochondria, as well as the promotion of the activation of CASP3. Additionally, a reduction in the protein expression of cyclinE1, cyclin-dependent kinase 2, and cyclinD1 in HeLa cells resulted in the cell cycle arresting at the G0/G1 phase [44] (Table 2). 

### 3.4. Ovarian Cancer

The cells of human ovarian cancer (A2780) were treated with hesperidin at various doses (0, 0.1, 1, and 10 µM). Ovarian cancer cells were less viable after hesperidin therapy in a way that was dependent on both time and dose. Hesperidin (1 as well as 10 µM) treatment resulted in a significant reduction in cell viability. Furthermore, these cancer cells meaningfully underwent apoptosis when exposed to 1 and 10 µM hesperidin [83] (Table 2).

### 3.5. Endometrial Cancer

The proliferative potential of hesperidin was measured through incubation with various doses of hesperidin at different time periods. In contrast with untreated cells, endometrial cancer cells subjected to hesperidin showed time- and dose-dependent reductions. Additionally, after incubating for time periods (24–72 h), the growth inhibition ratio was assessed, and it was discovered that hesperidin had time- and dose-dependent growth inhibition effects on endometrial cancer cells. In order to determine whether hesperidin had any potential apoptotic effects on these cancer cells, changes in CASP3 activation were observed in this study. The results of 48 h of hesperidin (50 µM) treatment on ECC-1 cells showed a 1.5-times rise in the percentage of CASP 3 in comparison with untreated cells [59] (Table 2).

### 3.6. Leukemia

A determination was carried out to investigate if apoptosis caused by hesperidin was caspase-dependent after hesperidin demonstrated a higher rate of apoptosis in NALM-6 cells. NALM-6 cells received hesperidin for 24 h at increasing concentrations (10–100 μM). The results showed that hesperidin treatment of the cells elevated the cleavage of caspase-9 and caspase-3 in a way that was dependent on concentration. In addition, hesperidin raised the expression of the Bax protein, whereas the Bcl-2 protein expression meaningfully reduced by the treatment of hesperidin in a dose-dependent manner. The results also showed that hesperidin for 48 h meaningfully decreased the incidence of XIAP within the NALM-6 cells [84] (Table 2). 

In KG1a cells, the pro-apoptotic potential of hesperidin was studied. Hesperidin treatment led to changes in the morphology of apoptotic cells and an increase in CASP3 activity. The findings exhibited that hesperidin treatment meaningfully decreased the expression of the antiapoptotic gene, including Bcl2 and survivin, and that treatment significantly increased the expression of the pro-apoptotic genes Bax and the cell cycle regulator p21 compared with the control group. These results indicated that hesperidin may be an effective apoptosis initiator and an appealing option in the therapy of acute myeloid leukemia [85]. 

According to the findings of another study, hesperidin was reported to be the most toxic to K562 cell lines. Hesperidin has been found to have a 24 h LC50 of 13.51 μg/mL as well as a 48 h LC50 of 10.41 mg/mL against K562 cells. The K562 cells were then exposed to concentrations of 40, 20, and 10 mμg/mL of these compounds for 3, 2, and 1 h, respectively. In Mcl-1 gene expression, hesperidin showed a hermetic effect in different dosages and incubation times [86] (Table 2). 

### 3.7. Liver Cancer

HepG2 cells were subjected to treatment within an invasion chamber using acetaldehyde and hesperidin to evaluate the effect of hesperidin on cell invasion caused by acetaldehyde. Acetaldehyde increased the migration of HepG2 cells 6-fold through Matrigel-coated filters. Hesperidin substantially lowered the percentage of HepG2 cells. Additionally, hesperidin effectively inhibited MMP-9 activation in HepG2 cells after acetaldehyde significantly promoted it. Following acetaldehyde treatment, MMP-9 expression was increased. In contrast, this expression was suppressed due to hesperidin in a manner that was dependent on concentration. Additionally, acetaldehyde-induced IB phosphorylation was suppressed by hesperidin (50 µM) in a way which was linked with the amount of hesperidin [87]. 

Hesperidin prevented both the released and cytosolic forms of matrix metalloproteinase (MMP)-9 within HepG2 cells, as well as cell invasion established by 12-O-tetradecanoylphorbol-13-acetate (TPA). The mRNA level of matrix metalloproteinase-9 that was induced by TPA was significantly reduced by hesperidin. Hesperidin has been reported to prevent the transcription of matrix metalloproteinase-9 by stifling the activity of NF-kB and AP-1. Furthermore, hesperidin inhibited AP-1 activity caused by TPA by suppressing p38 kinase phosphorylation and c-Jun N-terminal kinase signaling pathways. TPA-stimulated NF-kB translocation into the nucleus was also suppressed through NF-kB inhibitory signaling mechanisms.

The Hepatocellular carcinoma group showed a noteworthy increase in Cyclin D1, β-catenin, Wnt3a, and Wnt5a (wingless-related integration sites 3a and 5a) gene expressions and corresponding protein levels [68]. Hesperidin expressively inhibited thioacetamide-activated Wnt3a/β-catenin as well as Wnt5a pathways. Additionally, hesperidin employed a hepatoprotective effect via meaningfully improving inflammation, oxidative imbalance, serum alanine transaminase (ALT), and aspartate transaminase (AST) activities, liver function parameters, and albumin level. As a conclusion, hesperidin has been demonstrated to exert a prophylactic consequence towards hepato-carcinogenesis by restricting the induction of both canonical and non-canonical Wnt mechanisms [88] (Table 2). 

Hesperidin treatment of HepG2 cells resulted in the activation and enhancement of caspase-9, -8, and -3 activities. In addition, hesperidin treatment resulted in a dose-dependent reduction in Bcl-xL protein levels and increased concentrations of Bax, Bak, and tBid. Hesperidin causes apoptosis in human HepG2 cells via both the death receptor and mitochondrial pathways [89]. Hesperidin causes paraptosis-like cell death in HepG2 cells when ERK1/2 is activated, according to data from another study. This finding thus suggests that hesperidin-encouraging paraptosis may provide a different approach to treating human liver carcinoma [69].

### 3.8. Prostate Cancer

A flow cytometry technique was employed to determine the growth inhibitory potential of hesperidin. The findings demonstrated that as hesperidin concentration increased, the proportion of G2/M phase DU145 prostate cancer cells elevated proportionally. Furthermore, a linear relationship between the increasing hesperidin doses and the proportion of late apoptotic prostate cancer cells showed a significant increase. Under hesperidin administration, the concentrations of LDH release were found to increase considerably in a way that was related to concentration. Together, the findings support the hypothesis that hesperidin induces G2/M phase cell cycle arrest along with prostate cancer cell death similar to necrosis, which has been identified as a decline in their growth and proliferation. Additionally, it was found that at all of the hesperidin doses used, the ROS levels were significantly higher. When 20 µM hesperidin was used to expose DU145 cells, the ROS percentages proved to be approximately 2-fold higher. Analysis of the mitochondrial membrane potential (MMP) revealed that the MMP of cancer cells from the DU145 strain meaningfully decreased as the hesperidin concentration increased [90]. 

According to a new investigation, the relative cell distribution of the subG1 phase gradually increased as a consequence of dosage treatment with hesperidin at multiple concentrations, including 0, 5, 10, and 20 µM, indicating the beginning of the apoptotic process. The treatment of PC3 and DU145 cells with hesperidin (20 µM) decreased the immunofluorescence intensity of PCNA. In each cell line (PC3 and DU145), the relative fluorescence intensity dropped by more than 50%. These findings show that hesperidin governs the distribution of the cell cycle as well as inhibits the expression of markers associated with proliferation in cells of prostate cancer. In addition, JC-1 green monomers in PC3 and DU145 cells were raised following hesperidin therapy in a manner that was dependent on dose [91] (Table 2).

### 3.9. Renal Cancer

An investigation was conducted to determine the therapeutic interventions of hesperidin on rat renal carcinoma that were initiated by DEN administration and were endorsed by Fe-NTA. The ability of hesperidin to prevent cancer was assessed in relation to renal function, antioxidant activities, PGE2 levels, and the expression of VEGF and COX-2. Hesperidin upgraded renal function and restored renal antioxidant enzymes. It also demonstrated its effectiveness in decreasing DEN and Fe-NTA-induced lipid peroxidation. Hesperidin was also investigated for lowering PGE2 levels and for its ability to downregulate the expression of VEGF and COX-2. In histological studies, it was noticed that hesperidin had the ability to protect against both DEN and Fe-NTA-induced kidney damage [29]. 

Fe-NTA administration significantly exacerbated renal lipid peroxidation, wiped out renal antioxidant defense, and raised the levels of blood urea nitrogen (BUN), kidney injury molecule-1 (KIM-1), and creatinine. But concurrent pretreatment with hesperidin restores their levels in a dose-dependent manner. Further, it was discovered that hesperidin increased caspase-9, caspase-3, and bax expression while lowering bcl-2, NF-κB, inducible nitric oxide synthase (iNOS), tumor necrosis factor (TNF)-α, and PCNA expression. These findings provide compelling proof that hesperidin is an effective chemopreventiveagent against renal carcinogenesis [92].

### 3.10. Gall Bladder

Current statistics show that gallbladder cancer can kill 170,000 people annually, accounting for 1.7% of all cancer-related deaths, despite its size being less than 2 cm [93]. After hesperidin exposure to gall bladder carcinoma cells, significant modifications in the appearance of cancer cells were recorded. The results clearly showed that hesperidin promoted nuclear condensation in these cancer cells in a way that was dependent on its amount. Apoptotic cells are identifiable by condensed as well as fragmented nuclei. Bright-blue fluorescence and condensed nuclei staining in Hoechst 33342 were used to show apoptosis caused by hesperidin in treated cells. These results strongly indicate that hesperidin causes apoptosis in gallbladder carcinoma cells in a dose-dependent manner. Apoptotic induction in gall bladder carcinoma cells was also evaluated using annexin V-FITC/PI double-labeled flow cytometry following hesperidin therapy. Since the percentage of cells undergoing apoptosis elevated noticeably in a way dependent on concentration, identical consequences were achieved. Additionally, a dose-dependent increase in the activation of CASP3 and cell cycle arrest at G2/M phase was observed. All of these results suggest that hesperidin may have anticancer effects that could be used to treat gallbladder carcinoma [52] (Table 2).

### 3.11. Urinary Bladder Cancer

A study using a mouse model was conducted to examine the chemo-preventive potential of hesperidin as well as diosmin on urinary bladder cancer caused by N-butyl-N-(4-hydroxybutyl) nitrosamine (OH-BBN). A study was conducted using various animal groups, and OH-BBN (500 ppm) was administered for 6 weeks to all groups (1–7). At the commencement phase, groups 2 to 4 received diets with the experimental drugs (group 2, 1000 ppm diosmin only; group 3, 1000 ppm hesperidin alone; group 4, 900 ppm diosmin in addition to 100 ppm hesperidin together) for the full 8 weeks. Groups 5 to 7 received these diets, respectively, for the full 24 weeks of the post-initiation phase. When the experimental substances were administered throughout both phases, either individually or together in combination, bladder carcinoma and pre-neoplasia incidences were significantly reduced. The AgNOR count and BUdR-labeling index for different bladder lesions were substantially lowered by the dietary administration of these drugs, including hesperidin and diosmin. According to these results, hesperidin and diosmin could be able to successfully prevent bladder cancer both individually and together [94].

### 3.12. Malignant Pleural Mesothelioma 

MSTO-211H cells were subjected to hesperidin at a range of quantities (0–160 µM) to test the therapeutics of hesperidin on the morphology of cells in malignant mesothelioma cells. The outcomes displayed that following either a 48 h hesperidin treatment or no treatment, MSTO-211H cells contracted in size and modified into a round cell shape. Further, the effect of hesperidin on the apoptotic death of MSTO-211H cells was investigated. The results of the study revealed that mesothelioma cells treated with hesperidin exhibited greater nuclear condensation and fragmentation in contrast to the control. The sub-G1 population was increased in MSTO-211H cells by hesperidin [45] (Table 2).

### 3.13. Gastric Cancer

Hesperidin reduced the viability of the GC cells slowly and in a concentration-dependent way, demonstrating that hesperidin had suppressive efficacy on the proliferating capacity of GC cells. Moreover, in order to examine whether hesperidin prevented the proliferation of gastric cancer AGS cells via encouraging apoptosis, the results presented showed that with the increase in the duration of exposure to hesperidin, the detected green fluorescence intensity slowly enhanced, representing that the degree of apoptosis in AGS cells increased slowly. Moreover, morphological changes in apoptosis, for example, cell shrinkage and decrease in cell density, were noticed. With the constant enhancement of the duration of hesperidin treatment, the number of apoptotic cells increased slowly, and the apoptosis touched its highest level when the treatment duration was 24 h. In addition, hesperidin treatment was directed to slow down and induce a time-dependent enhancement in ROS. Furthermore, apoptosis was meaningfully reduced in the group treated with hesperidin and NAC, compared to the group treated with hesperidin only [49].

### 3.14. Oral Cancer

With a substantial rate of death, oral cancer has become among the top six cancers globally and is a subtype of head and neck squamous carcinoma [95]. Hesperidin therapy was found to substantially decrease the mean cell viability percentage values in a manner that was time- and dose-dependent. Furthermore, the morphology of HN6 and HN15 resulting from exposure to hesperidin at 50 or 200 µM was examined. From the culture vessel, certain HN6 and HN15 cell lines have been identified and taken out. Hesperidin treatment decreased the number of colonies in a dose-dependent manner. However, substantial reductions in the mean percentage values of the colonies were obtained after treatment with 50 µM of hesperidin in both the HN6 and HN15 cell lines. This shows that hesperidin treatment could stop both oral cancer cell lines from proliferating. The outcomes of the cell invasion test also show that therapy with hesperidin hindered a substantial increase in the mean percentage values of cell invasion after IFN intervention across HN6 and HN15 [58]. 

An investigation was carried out to examine the cancer-fighting potential of hesperidin compared with doxorubicin against HEp-2 (laryngeal carcinoma cells). Hesperidin was reported to have an antiproliferative effect on HEp-2 cells that becomes stronger over time. For both of the investigated drugs, gene profile analysis revealed an extremely noteworthy reduction in the turning on (expression) of antiapoptotic Bcl-2 gene and an extremely substantial rise in the case of p53 (tumor suppressor gene) expression. In the HEp-2 cell line, hesperidin reduced cancer cell viability through cell cycle arrest and apoptotic mechanisms, demonstrating possible cancer-fighting properties [96]. 

### 3.15. Brain Cancer

The U-87 cell line was employed to determine the potential efficacy of hesperidin in the treatment of brain glioblastoma tumors. According to the results, treatment with hesperidin (10 µM and 25 µM) resulted in a disturbance of the membrane potential of mitochondria (46% at 10 µM and 28% at 25 µM) in regard to a variety of caspase pathways. In a 48 h incubation period, hesperidin (10 µM) resulted in 32.6% of alive cells, and hesperidin at 25 µM led to 25% of alive cells. According to the findings of this research, hesperidin is suggested to have an antiproliferative and antiapoptotic effect [97] (Table 2). 

### 3.16. Bone Cancer

According to a significant osteosarcoma study, hesperidin had time- and concentration-dependent cytotoxic effects at various doses (0–200 µM). When the doses of hesperidin were raised from 50 to 100 µM, a sharp, significant increase in the cytotoxic potential was observed. Furthermore, MG-63 cells started to undergo apoptosis as hesperidin doses increased. In cells exposed to 5, 50, and 150 M of hesperidin, the proportion of apoptotic cells grew from 4.7% in untouched cells as a control to 17.9, 34.6, and 68.3%, respectively. Early and late apoptosis were also observed to be induced. Additionally, the findings of this study showed that hesperidin triggered cell cycle arrest at the G2/M stage at higher doses. In addition, the G2/M cell percentage rose from 29.2% within untreated cells to 35.1, 46.3, and 72.4% in cells treated with 5, 50, and 150 µM hesperidin, respectively. The G0/G1 cell population decreased concurrently as the hesperidin concentration increased from 0 to 150 M [51] (Table 2). 

### 3.17. Melanoma

The survival of B16BL6 cells was investigated using multiple concentrations (50, 25, and 12.5 µg/mL) of hesperidin in order to investigate the increase in the tumor inhibition of growth in correlation with dose. In contrast to intact hesperidin, the 150 kGy gamma-irradiated hesperidin produced a more potent dose-related suppression of tumor cell growth. The irradiated hesperidin had the same effect on normal cells in terms of slowing down growth as intact hesperidin. These findings strongly implied that cancerous cells were significantly more sensitive to 150 kGy. The possibility of preventing B16BL6 cell growth through treatment with irradiated hesperidin was also explored. The irradiated hesperidin was given to B16BL6 cells at a concentration of 50 µg/mL. In comparison to the intact hesperidin treatment, the treatment of B16BL6 cells with irradiated hesperidin significantly changed the appearance of those cells. In addition, the relationship between ROS production and the apoptosis caused by irradiated hesperidin was also examined. The findings documented that irradiated hesperidin caused an increased improvement in comparison to intact hesperidin that was dependent on both time and dose for the production of ROS by B16BL6 cells [98]. 

### 3.18. Esophageal Cancer

The effects of the flavonoids hesperidin and diosmin on esophageal carcinogenesis brought on by N-methyl-N-amyl nitrosamine (MNAN) were investigated both during and after the initial phases. Groups of animals were fed diets containing 1000 ppm diosmin and 1000 ppm hesperidin, as well as diets containing both compounds (900 ppm diosmin and 100 ppm hesperidin) for 13 weeks, commencing 7 days prior to the MNAN dosing, before switching to the basal diet. This was conducted to examine the modifying effects of the “initiation” treatment of test compounds. The groups given MNAN and a basal diet were switched to the experimental diets containing diosmin, hesperidin, or diosmin combined with hesperidin at 1 week after the stop of MNAN injection and maintained on these diets for 7 weeks in order to examine the modifying effects of ‘post-initiation’ treatment of these compounds. The findings suggest that the administration of diosmin and hesperidin, either separately or together, is beneficial in the avoidance of the onset of esophageal cancer induced by MNAN if given throughout the beginning stage. This protection may be linked to the reduction in the greater proliferation of cells in the esophageal mucosa caused by MNAN [99].

### 3.19. Colon Cancer

In a recent study, the chemo-preventive potential of hesperidin in colorectal cancer (CRC) caused by 1, 2-dimethylhydrazine (DMH) was investigated in a recent study. The 2-dimethylhydrazine (DMH) group showed a significant rise in MDA and NO levels as well as the expression of the activin A protein and gene. The SMA- and MAD-related protein 4 (Smad4), GSH, and superoxide dismutase (SOD) levels in tissues all decreased significantly, as did the expression of their corresponding genes and proteins. Hesperidin treatment significantly increased the expression of the activin A and Smad4 genes in both the 1,2-dimethylhydrazine (DMH) plus hesperidin and the DMH after hesperidin groups. Hesperidin’s ability to prevent colorectal cancer by modulating Smad4 and activin A signaling in vivo was confirmed by this study [100]. 

Another study reported that the treatments with hesperidin meaningfully prevented the number as well as multiplicities of azoxymethane-induced ACF as well as the incidence of tumor. Hesperidin has been documented to enhance antioxidant status and decrease oxidative stress parameters. In addition, the PCNA index fell after hesperidin administration. Inflammation was clearly reduced by hesperidin treatments by downregulating NF-kB and its target molecules, COX-2 and iNOS [101]. 

In mice with tumors, the effect of hesperidin on cyclophosphamide’s antitumor activity was examined. Prior to the administration of cyclophosphamide, hesperidin at a concentration of 200 mg/kg raised white blood cell counts. After receiving cyclophosphamide injections, this notable ability to protect was reported on the 4^th^ and 7^th^ days. Hesperidin and cyclophosphamide co-administration was found to significantly reduce/prevent the tumor growth delay caused by cyclophosphamide in mice bearing colon cancer. These results demonstrated that hesperidin interferes with the antitumor activity of cyclophosphamide. The estrogen receptor was not involved in the growth of the CT-26 tumor. These findings imply that hesperidin-containing foods, like citrus, may reduce the efficacy of cyclophosphamide in the therapy of colon cancer patients [102]. 

At 100 µM, hesperidin was reported to decrease cell viability. Further, the cell death induced by hesperidin was found to be linked with apoptotic features. Additionally, hesperidin therapy decreased B-cell CLL/lymphoma 2 (BCL2) mRNA expression and increased the expression of BCL2-associated X protein (BAX). With hesperidin treatment, the expression as well as the activity of the key apoptotic factor caspase3 (CASP3) were documented to be substantially increased. Hesperidin was further shown to decrease the protein expression of pro-CASP3. On the contrary, hesperidin induced an increase in the level of active CASP3 [48].

**Table 2:** Role of hesperidin in cancer prevention and treatment.

Cancer	Types of Study	Dose Ranged	Findings	Refs.
Lung	In vitro, A549 cells	12.5 μM and 25 μM	The percentage of apoptotic nuclei after treatment with hesperidin increased enormously to 37% and 59%, respectively, as discovered by nuclear condensation and fragmentation. Hesperidin-treated cells resulted in a noteworthy increase in DNA fragmentation appearance.	[73]
	In vitro, A549 and NCI-H358	5–50 μM	There have been notable, concentration- and time -dependent declines in cell proliferation and rises in cytotoxicity.	[72]
	In vitro, A549 and NCI-H460	10, 25, 50 μM	Hesperidin treatment inhibited Bcl-2 mRNA expression in cancer cells, and this expression fell as the concentration of hesperidin gradually increased.	[74]
Breast cancer	In vivo, Rats	200 mg/kg	Hesperidin has a protective effect against experimentally induced breast cancer that seems to be related to a decrease in Ki67 expression.	[78]
	In vitro, MDA-MB231	10 to 50 µM	Treatment with hesperidin (10 to 50 µM) significantly reduced the expression of PD-L1 along with various proteins involved in signaling pathways.	[79]
	In vivo, Rats	30 mg/kg/body weight	Noteworthy reductions in the status of antioxidant levels and alterations in the drug metabolizing enzymes were found in genotoxin DMBA treated animals. Interestingly, these changed levels were meaningfully revered back to near normal in hesperidin given animals.	[80]
Cervix cancer	In vitro, HeLa	0,40,80,160 µM	HeLa cancer cells showed meaningfully increased caspase-3 in hesperidin-treated cells.	[44]
Ovarian	In vitro, A2780	1 and 10 µM	At hesperidin concentrations (1 and 10 µM), A2780 cell viability was meaningfully reduced and induced apoptosis in these cancer cells.	[83]
Endometrial cancer	In vitro, ECC-1	50 μM	Caspase 3 levels on ECC-1 cells were 1.5 times higher after 48 h of treatment with 50 M hesperidin when compared to untreated cells.	[59]
Leukemia	In vitro, NALM-6	10–100 μM	Hesperidin treatment of the cells led to a concentration-dependent improvement in caspase-3 and caspase-9 cleavage.	[84]
	In vitro, K562	10, 20, and 40 μg/mL	Hesperidin was the cytotoxic compound against K562 cell lines.	[86]
Liver	In vitro, HepG2	50 µM	The increased number of HepG2 cells was meaningfully prevented by hesperidin. Additionally, hesperidin effectively inhibited MMP-9 activation stimulated by acetaldehyde.	[87]
	In vitro, HepG2	50 µM	In HepG2 cells, hesperidin inhibited the secreted and cytosolic forms of MMP-9 and reduced cell invasion caused by TPA (12-O-tetradecanoylphorbol-13-acetate).	[68]
	In vivo, Rats	150 mg/kg/day	Hesperidin meaningfully inhibited thioacetamide-activated Wnt3a/β-catenin as well as Wnt5a pathways.	[88]
Prostate cancer	In vitro, PC3 as well as DU145	0, 5, 10, and 20 µM	The relative cell distribution of the subG1 phase progressively improved after hesperidin treatment, which was dose-dependent.	[91]
Renal cancer	In vivo, Rats	100, and 200 mg/kg/day	Hesperidin reduced the amount of lipid peroxidation treatment caused by Fe-NTA and DEN and enhanced renal activity.	[29]
Gall bladder cancer	In vitro, GBC	200 µM	Therapy with hesperidin substantially lowered the proliferation of gall bladder carcinoma cells.	[52]
Urinary bladder cancer	In vivo, Mice	1000 ppm hesperidin (4900 ppm diosmin along with 100 ppm hesperidin)	Hesperidin and the flavonoids diosmin are potent inhibitors of chemical carcinogenesis of the bladder, both separately and in combination.	[94]
Malignant pleural mesothelioma	In vitro, MSTO-211H	0–160 µM	When MSTO-211H cells were left untreated or given hesperidin, the size of the cells shrank, and they assumed a round cell shape.	[45]
Gastric cancer	In vitro, AGS	20 μM	Treatment with hesperidin focuses on a gradual, time-dependent increase in ROS.	[49]
Oral cancer	In vitro, HN6 and HN15	10–50 μM	Hesperidin treatment decreased the viability of cells in a way that was both time- and dose-dependent.	[58]
Brain cancer	In vitro, U-87	10 uM and 25 uM	When given hesperidin (10 µM and 25 µM), the potential of the mitochondrial membrane was disrupted.	[97]
Bone cancer	In vitro, MG-63	5, 50 and 150 µM	The onset of apoptosis was triggered by increasing hesperidin doses. Hesperidin treatment increased the percentage of apoptotic cells and induced early and late apoptosis.	[51]

## 4. Synergistic Effects of Hesperidin with Anticancer Drugs/Natural Compounds

**Figure 4:**
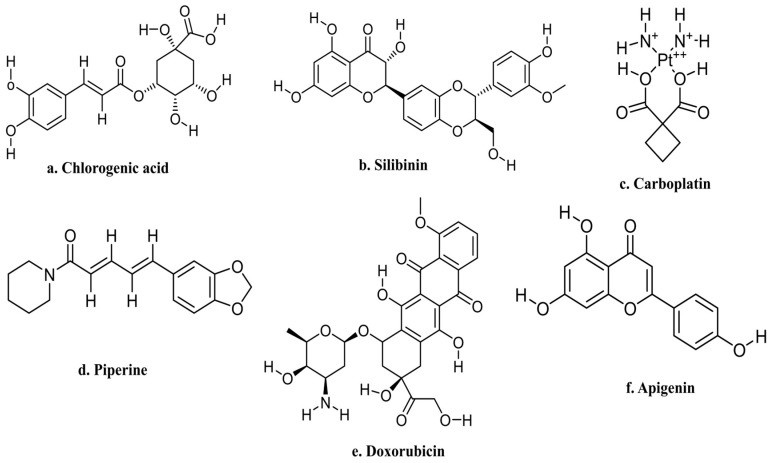
Structure of various substances. (**a**) Chlorogenic acid; (**b**) silibinin; (**c**) carboplatin; (**d**) piperine; (**e**) doxorubicin; (**f**) apigenin.

The combination of anticancer drugs with different types of natural compounds or their biomolecules has proven its involvement in the protection of cancer by enhancing the efficacy of anticancer drugs and reducing adverse complications. In this regard, hesperidin shows synergistic effects with anticancer drugs or other natural compounds in cancer treatment. According to a breast cancer study, the combination of hesperidin and chlorogenic acid (Figure 4a) can regulate the mitochondrial function and production of ATP in breast tumors (MCF-7) cells by acting on the estrogen receptor pathway. Finally, research exhibited the effectiveness of hesperidin as well as chlorogenic acid when used in conjunction with chemotherapy for breast cancer patients [103] (Table 3). 

According to an in vitro study, the IC50 values for hesperidin and silibinin (Figure 4b), respectively, were 50.12 µM and 16.2 µM, indicating their cytotoxic ability. Hesperidin, as well as silibinin, both showed synergistic potential, according to a Combination Index study, and they each decreased Cytarabine’s IC50 value by almost 5.9- and 4.5-folds, respectively. Due to their ability to cause antileukemic effects, both natural compounds may be used alone as well as together with other chemotherapeutic agents as part of acute myeloid leukemia (AML) therapy [104] (Table 3). A549 and NCI-H460 cells demonstrated a mutually beneficial outcome together with the drugs and provoked the maximum apoptosis in the hesperidin along with carboplatin (Figure 4d) treatment group. Hesperidin and carboplatin were shown to inhibit cell proliferation in the results. In the hesperidin combined with carboplatin treatment group, the rates of A549 and NCI-H460 cell proliferation were at their lowest. Drugs were affected by the combination in a synergistic manner. Hesperidin and carboplatin have been demonstrated to be particularly successful at preventing the invasion of NCI-H460 and A549 cells, according to the results of the analysis of cell invasion. The most considered inhibitory effect was seen when hesperidin and carboplatin were administered together. Additionally, in NCI-H460 and A549 cells, hesperidin and carboplatin increased the expression of p53. The group receiving treatment with carboplatin and hesperidin had the highest level of p53 expression. The apoptosis-related genes p21, Bax, and PUMA showed increased expression levels when exposed to either carboplatin or hesperidin. The highest levels of p21, PUMA, and Bax expression were seen in the group that received both hesperidin and carboplatin [74] (Table 3). 

Hesperidin, piperine (Figure 4d), and bee venom in a combined fashion improved the anticancer potential of tamoxifen and could be used as a safe adjuvant/vehicle to tamoxifen in the treatment of breast cancer [105] (Table 3). Another test was conducted to see if hesperidin, when combined with doxorubicin (Figure 4e), could conquer resistance to the doxorubicin in MCF-7-resistant doxorubicin cells (MCF-7/Dox) in regard to cytotoxicity, apoptosis, and the expression of P-glycoprotein (Pgp).

Another experiment was conducted to examine the ability of hesperidin to overcome doxorubicin resistance in MCF-7-resistant doxorubicin cells (MCF-7/Dox) in terms of cytotoxicity, apoptosis, and the expression of P-glycoprotein (Pgp) when combined with doxorubicin. On MCF-7/Dox cells, hesperidin by itself demonstrated cytotoxic potential via an IC50 value of 11 µmol/L. Hesperidin and doxorubicin combined therapy had an antagonistic and addictive effect. Apoptotic induction was not increased by hesperidin but reduced the P-glycoprotein expression level when combined with doxorubicin at a low concentration [106]. Furthermore, hesperidin (5, 50, and 100 µM) improved the cytotoxic potential of doxorubicin compared with doxorubicin individually. The synergistic use of 100 µM hesperidin as well as 200 nM doxorubicin demonstrated excellent cytotoxic activity. MCF-7 cells underwent apoptosis after receiving a treatment that consisted of doxorubicin and hesperidin [107] (Table 3). 

Hesperidin and apigenin (Figure 4f) both have an impact on MCF-7 breast cancer cells when combined with doxorubicin. It was determined that flavonoids enhanced doxorubicin’s potential for cytotoxicity despite the fact that they actually decreased the drug’s capacity to cause damage from oxidative stress. Finally, both apigenin and hesperidin increase the cytotoxic role of doxorubicin on breast cancer cells [108].

**Table 3:** Synergistic effects of hesperidin with other natural bioactive molecules.

Caner Type	Hesperidin/Anticancer Therapy/Natural Bioactive Molecules	Findings of Investigations	Refs.
Breast	Chlorogenic acid and hesperidin	Combination therapy with hesperidin and chlorogenic acid has a synergistic effect that might regulate ATP production and mitochondrial activity through the estrogen receptor mechanism in cancer cells.	[103]
Breast	Hesperidin, piperine, bee venom/ tamoxifen	Tamoxifen’s ability to fight cancer is enhanced by the synergistic effects of hesperidin, piperine, and bee venom.	[104]
Lung	Hesperidin plus carboplatin	Hesperidin in addition to carboplatin therapy caused the greatest degree of apoptosis in lung cancer cells and had a synergistic effect on the other drugs. Hesperidin + carboplatin treatment group had the lowest rates of cell proliferation.	[74]
breast	Hesperidin and doxorubicin	In comparison to doxorubicin alone, hesperidin increased doxorubicin’s cytotoxic potential. The combination of doxorubicin and hesperidin demonstrated excellent cytotoxic activity.	[106]
leukemia	Hesperidin and silibinin	Hesperidin and silibinin both demonstrated synergistic potential and decreased cytarabine’s IC 50 value by 5.9- and 4.5-folds, respectively.	[107]
Breast	Hesperidin, apigenin, and doxorubicin	Hesperidin and silibinin both demonstrated synergistic potential and decreased cytarabine’s IC 50 value by 5.9- and 4.5-folds, respectively.	[108]

## 5. Approaches to Improve the Efficacies of Hesperidin 

The use of hesperidin in treating a variety of diseases has been proven in numerous experimental studies. Numerous investigations have shown that hesperidin has a lower bioavailability because of its very low aqueous solubility, which may significantly restrict its absorption [109,110,111]. Numerous techniques based on nano-formulations have been developed in order to address the problems of poor solubility, quick elimination, and metabolism, and their role in the prevention of cancer has been investigated. To ascertain whether hesperidin nanoparticles and/or imatinib mesylate individually or together increased the cancer protecting potential, a significant study was carried out. It also examined whether nanoencapsulation could be used to reduce the cardiotoxicity of imatinib mesylate in mice with solid Ehrlich carcinoma. Hesperidin and imatinib mesylate were added to PLGA (poly(lactic-co-glycolic acid) polymer).

Solid Ehrlich carcinoma-bearing mice have been developed as a model for breast cancer brought on by experimentation. When compared to control groups that received only conventional treatment, the groups treated with nano-imatinib mesylate and/or nano-hesperidin showed a notable decline in weight, tumor volume, hematological markers, cardiac markers, and downregulation of the tumor MDR-1 gene [112]. Hesperidin delivery using a targeted nanohybrid carrier system was investigated. The process of desolvation along with ionic-gelation was implemented to create a casein–calcium–ferrite nanohybrid carrier. Hesperidin was first capsuled in a carrier, and then progesterone, the directing ligand, was conjugated via an activated ester procedure. Maximum hesperidin encapsulation in the carrier was achieved through Taguchi optimization at 89.54%. Better drug encapsulation and magnetic drug delivery were produced by the merger of superparamagnetic calcium ferrite nanoparticles. The drug release behavior of the carrier was stimuli-responsive, with better stability and a greater release of drug applications that were beneficial to treating cancer. The biocompatibility of the formulation was verified by a cell viability assay using L929 fibroblast cells. The progesterone-conjugated carrier’s particular recognition and aimed chemotherapy improved the cytotoxic activity of CHD against SKOV-3 ovarian and MDA-MB-231 breast cancer cells, leading to a noTable 30-fold decrease in the half-maximal inhibitory concentration (IC50) values [113]. The cytotoxic potential of hesperidin loaded on gold nanoparticles (Hsp-AuNPs) against MDA-MB-231 (human breast cancer) cells was investigated. The findings showed a noteworthy reduction in the suppression of the growth and proliferation of treated cells compared with normal human breast epithelial cells. Hsp-AuNPs administration did not cause any histopathological changes or damages in the liver, lung, spleen, or kidney. These nanoparticles enhanced the functional behavior of macrophages in mice with Ehrlich ascites tumors [114]. Two nanoformulations, i.e., hesperetin-TPGS (D-atocopheryl polyethylene glycol 1000 succinate) micelles and hesperetin-phosphatidylcholine complexes, increased the aqueous solubility, antioxidant activity, and oral absorption of hesperetin and led to 16.2- and 18.0-fold enhancements in the in vitro antioxidant activity and in vivo oral absorption of hesperetin [115]. According to the research conducted on HEp-2 cells, bioformulated hes-PLGA-NPs were found to be more effective than native hesperidin and were suggested to be a strong anticancer candidate [116]. In another study, hesperidin-PLGA-poloxamer 407 was successfully prepared to reduce and avoid issues related to hesperidin absorption, improving its stability and bioactive potential. The finding showed that nanohesperidin can be used as a driving formulation for new chemotherapeutic agents in clinical trials [117].

## 6. The Concentration of Hesperidin in Various Fruits and Vegetables 

Many different kinds of fruits and vegetables contain natural products, including flavonoids that have been reported to have the potential to fight against several diseases [118,119]. Currently, dietary intake continues to be the main way to obtain hesperidin. Hesperidin is present in some fresh fruits and vegetables in significant amounts. The most popular dietary sources of hesperidin are citrus fruits and citrus juices. The concentration of hesperidin in various fruits and vegetables is provided in Table 4. 

## 7. Clinical Studies of Hesperidin

Very few studies have been performed on human subjects to evaluate the role of hesperidin in disease management, including cancer. Following the ethical principles described in the 1975 Helsinki Declaration and its revisions, a clinical trial randomized to nutritional intervention was conducted [130]. The goal of this trial was to explore the metabolic profile of dietary polyphenols in normal and malignant glandular breast tissues from individuals with recently diagnosed breast cancer. 

The ClinicalTrials.gov Identifier of this study is NCT03482401. Patients, who provided written consent (28 patients out of a total 43 patients), were divided at random into two groups. The polyphenol group patients took capsules from the time of diagnosis until the time of surgery. However, the control group took no capsules. The capsule was a nutritional supplement high in polyphenols, particularly simple phenolics like hydroxytyrosol and polyphenols, including procyanidins, hesperidin, eriocitrin, curcumin, resveratrol, punicalagin, and ellagic acid. Methylxanthines, theobromine, and caffeine were additionally present in capsules with cocoa extract. 

By using UPLC-ESI-QTOF-MS, a total of 101 metabolites were found in urine, 69 in plasma, 39 in healthy (NT) tissues, and 33 in malignant (MT) tissues. Eight patients served as controls and did not take any extracts. In MT and NT, phenolic-derived metabolites are primarily glucuronidated and/or sulfated. In MCF-7 breast cancer cells, metabolites that have been found in breast tissues have no antiproliferative or estrogenic/antiestrogenic effects [130] (Table 5).

## 8. Conclusions

The choices we make regarding our way of life and environment have a big impact on our chances of developing cancer. If there are several unavoidable risk factors, we can alter our lifestyles by reducing carbohydrates, smoking, etc., including natural products, bioactive molecules in our diet, and taking environmental action to lessen the risk of cancer. Hesperidin is a bioflavonoid and is considered a potent bioactive compound with a wide range of effective pharmacological potential due to its antioxidant and anti-inflammatory potential. Additionally, anticancerous potential has been proven through the modulation of signal transduction pathways, including cell cycle, apoptosis, angiogenesis, activating protein-1, ERK/MAPK, and signal transducer and the activator of transcription. The synergistic effect of hesperidin in cancer management has been described in combination with cancer agents or natural products. Hence, hesperidin might be very beneficial in developing a potential therapeutic anticancer strategy. The current study demonstrates that hesperidin is extremely beneficial in preventing or treating several cancer types, including lung cancer, colon cancer, prostate cancer, etc. However, the current analysis demonstrates that hesperidin is particularly effective in ovarian cancer lines (in vitro study) and in rats with breast cancer (in vivo study) when taking into account the dose of hesperidin with respect to efficacy in different cancer types. Hesperidin significantly reduced cell viability and induced apoptosis in ovarian cancer cells (A2780 cells) when used at concentrations of 1 and 10 µM, according to an in vitro study. Hesperidin, at a dose of 30 mg/kg/body weight, restored antioxidant levels and reversed changes in the drug metabolizing enzymes in rats with breast cancer (genotoxin DMBA treated rats). Hesperidin’s beneficial chemo-preventive effect, however, has only been observed in pre-clinical in vitro and in vivo cancer models. The results of combining the data showed that hesperidin’s anticancer effects involve multiple roles. Despite this, it is very difficult to determine the most effective doses for hesperidin’s anticancer effects in the human body due to insignificant clinical trials on its useful role. Therefore, detailed research with mechanisms of action as well as clinical trial-based studies are needed to explore the potential benefit of hesperidin in health management. Future research should, in our opinion, concentrate on improving hesperidin’s bioavailability and absorption, identifying the precise molecular mechanisms underlying its anticancer effects, determining the optimal doses for hesperidin clinical trials, and assessing the anticancer effects of hesperidin in cancer patients.

## Figures and Tables

**Table 4:** The concentration of hesperidin in various fruits and vegetables.

Source	Concentration of Hesperidin (Mean Value)	Refs.
*Sorbus tianschanica*	0.48 mg/100 g	[120]
*Citrus sinensis*	28.6 mg/100 mL	[121]
*Citrus clementina*	24.3 mg/100 mL	[122]
*Citrus clementina*	39.9 mg/100 mL	[123]
*Citrus deliciosa*	0.15 mg/100 mL	[121]
*Citrus limon*	20.5 mg/100 mL	[124]
*Citrus aurantifolia*	1.77 mg/100 mL	[122]
*Citrus sinensis*	0.2/g FW, 4.8 g/g FW	[125]
*Mentha piperita*	480.85 mg/100 g FW	[126]
*Allium fistulosum*	0.02 mg/100 g FW	[127]
*Cyclopia maculata*	11.82 g/100 g	[128]
*Citrus unshiu*	37.3 mg/100 g	[129]

**Table 5:** Details of clinical trial linked with hesperidin.

ClinicalTrials.gov Identifier	Disease/Condition	Intervention/Treatment	Title	Status
NCT03482401	Breast cancer	Dietary supplement: polyphenol	Disposition of Dietary Polyphenols and Methylxanthines in Mammary Tissues From Breast Cancer Patients	Completed

## Data Availability

Not applicable.

## Abbreviations

**Abbreviation**	**Full form**
PGE2	Prostaglandin E2
iNOS	Nitric oxide synthase
Fe-NTA	Iron nitrilotriacetate
DEN	N,N-diethylnitrosamine
COX-2	Cyclooxygenase-2
VEGF	Vascular endothelial growth factor
ROS	Reactive oxygen species
B[α]p	Benzo(α)pyrene
GSH	Glutathione
LDH	Lactate dehydrogenase
CCl_4_	Carbon tetra chloride
TBARS	Thiobarbituric acid reactive substances
CAT	Catalase
GPx	Glutathione peroxidase
SOD	Superoxide dismutase
Bid	BH3 interacting domain death agonist
CASP3	Caspase-3
PARP	poly (ADP-ribose) polymeraseas
Bax	Bcl-2-associated X protein
Bcl-xl	B-cell lymphoma-extra large
MAP Kinase (MAPK)	Mitogen-activated protein kinase
PD-L1	Programmed cell death ligand 1
STAT3	Signal transducer and activator of transcription
PI3K	Phosphoinositide 3-kinase
mTOR	Mammalian target of rapamycin
CDK-2	Cyclin-dependent kinase-2
AP-1	Activating protein-1
NF-kB	Nuclear factor kappa B
TPA	12-O-tetradecanoylphorbol-13-acetate
TEM	Transmission electron microscopy
ZEB2 mRNA	Zinc finger E-box binding homeobox 3 mRNA
MTT assay	3-[4,5-dimethylthiazol-2-yl]-2,5 diphenyl tetrazolium bromide assay
c-myc	Myelocytomatosis
PCNA	Proliferating cell nuclear antigen
MDMX	Murine double minute X
DMBA	7,12-dimethylbenz(a)anthracene
